# Cognitive deterioration in patients undergoing hemodialysis: how variations in age influence the development of new mechanisms and treatment approaches?

**DOI:** 10.3389/fnagi.2025.1645702

**Published:** 2025-09-08

**Authors:** Hongyu Jiang, Dongyuan He, Yue Hu

**Affiliations:** ^1^Blood Purification Center, Zhejiang Hospital, Hangzhou, China; ^2^Nephrology Department, Zhejiang Hospital, Hangzhou, China

**Keywords:** hemodialysis, children, elderly, end-stage renal disease, neurodevelopmental and cognitive impairment, high-dose hemodiafiltration

## Abstract

The mechanisms of age-related differences and innovative intervention strategies for cognitive dysfunction in hemodialysis patients are crucial for enhancing patient outcomes. This research thoroughly examined the varying pathological aspects of cognitive decline across different age groups. Children and adolescents experience heightened permeability of the blood–brain barrier during critical developmental phases, along with the disruptive effects of uremic toxins on neurotransmitters and synaptic plasticity, which result in diminished white matter integrity and abnormal functioning of the default mode network. Additionally, genomic variations, such as harmful CNVs, coexist with the central nervous system’s high plasticity and susceptibility. In contrast, elderly patients face cognitive impairment due to the combined effects of vascular diseases (like small vessel disease and impaired cerebral blood flow regulation) and Alzheimer’s-like pathology, exacerbated by dialysis-related hypotension, oxidative stress, and inflammation, which further contribute to reduced cerebral blood flow and neurodegeneration. Consequently, a life cycle-based layered intervention strategy is suggested: children should focus on safeguarding their neural development through collaborative gene–environment interventions and neural stem cell transplants, while elderly patients require standardized treatment for vascular diseases and comorbidities, including Alzheimer’s disease. Evidence indicates that incremental dialysis, low temperature dialysis, and high-dose hemodiafiltration can significantly reduce inflammation and oxidative stress markers, slow cognitive decline across all ages, and offer new insights for targeted nephrology management due to their universal effects. Future multi-center cohort studies are necessary to confirm the long-term advantages of age-specific interventions and to support the development of personalized precision treatment systems.

## 1 Introduction

The advancement and widespread use of blood purification technology have greatly extended the lifespan of patients with end-stage renal disease (ESRD); however, the rate of cognitive dysfunction linked to this condition continues to be elevated (Tian et al., 2022; Song et al., 2020). Patients undergoing hemodialysis experience considerable difficulties with executive function, attention, and episodic memory, and their rate of cognitive decline is 3 to 5 times quicker than that of the general elderly population (Kanbay et al., 2025; Zhang et al., 2024). This decline has a direct impact on their adherence to dialysis, the safety of their medications, and the burden on their families and social networks. What is even more alarming is that these patients experience a considerably poorer long-term outlook and are at an increased risk of disability and mortality (Gela et al., 2021; McAdams-DeMarco et al., 2018). Maintenance hemodialysis can result in different levels of cognitive decline in patients of all ages, including children, adolescents, adults, and the elderly. This decline may present as memory issues, reduced executive function, and slower information processing. Research has shown that cognitive impairment associated with hemodialysis is linked to factors like oxidative stress, inflammation, neurotoxins from uremia, and low blood pressure during the procedure (Lu et al., 2015; Lisowska-Myjak, 2014; Flythe et al., 2015). Current research frequently categorizes patients across various age groups as homogeneous entities, thereby neglecting the age-specific physiological and pathological differences that exist. It is essential to recognize that the pathogenesis of cognitive impairment can vary significantly across different life stages. In pediatric and adolescent populations, who are undergoing critical neural development and exhibit heightened blood–brain barrier permeability, exposure to uremic toxins may impede cognitive growth by disrupting neurotransmitter synthesis, synaptic plasticity, and other related processes. Conversely, in adult populations, particularly among the elderly, vascular disease is often identified as a primary underlying mechanism. Conditions such as atherosclerosis and cerebrovascular diseases—including white matter hyperintensities and microhemorrhages—along with compromised cerebral blood flow autoregulation during dialysis, contribute to recurrent hypoperfusion-related brain injuries. Elderly patients are particularly susceptible to the exacerbation of neurodegenerative diseases, such as those characterized by Alzheimer’s disease (AD)-like pathological changes and chronic inflammatory states. The interplay of uremic toxins and brain iron deposition may further activate glial cells, leading to accelerated tau protein phosphorylation and neuronal apoptosis (Zoccali et al., 2025). Given that the occurrence of AD among older adults is greater than that of other forms of dementia (Pérez Palmer et al., 2022), conversations regarding the cognitive effects of hemodialysis in the elderly primarily center around neurodegenerative diseases, particularly AD. It is important to highlight that contemporary research on mechanisms predominantly concentrates on a singular population and often lacks comparative analyses across different age groups. This “one size fits all” research approach may result in a limited applicability of intervention strategies. For instance, early prenatal diagnosis and preventive measures may be essential for children with neurodevelopmental disorders, whereas cognitive training for elderly individuals must consider the specific pathological characteristics associated with neurodegenerative diseases. In light of this, the present article systematically examines the pathophysiological differences in cognitive impairment between children and elderly patients, proposing a targeted intervention hypothesis grounded in the life cycle. This hypothesis suggests that cognitive decline can be mitigated through specific intervention strategies tailored to both childhood and elderly stages, including the optimization of toxin clearance methods (such as high-dose hemodiafiltration) and the regulation of hemodynamic stability (for example, through low temperature dialysis). This study not only provides a theoretical foundation for clinical hierarchical management but also establishes a basis for advancing the objective of “precision nephrology.”

## 2 Heterogeneity of age-related cognitive impairment mechanisms

Current research has established age as a significant risk factor for cognitive impairment in individuals receiving hemodialysis. A longitudinal cohort study conducted by Drew et al. (2017b) involving 314 elderly patients demonstrated that age serves as an independent predictor of the rate of decline in executive function. It is important to highlight that the Odagiri research team (Odagiri et al., 2011) conducted a controlled study involving 154 patients undergoing hemodialysis and 852 individuals from the general population. Their findings indicate that hemodialysis treatment interacts synergistically with age, and this combined effect may elevate the risk of cognitive impairment. These two studies have collaboratively developed a clinical evidence system addressing age-related cognitive impairment; however, the prevailing research paradigm exhibits notable limitations. Primarily, the majority of findings concentrate on the elderly demographic, specifically individuals aged 65 and older, leaving a significant gap in the investigation of age-specific mechanisms in middle-aged dialysis patients (ages 45–65) and pediatric patients. This selective bias may obscure the diverse characteristics of cognitive decline across different age groups. For instance, children may experience cognitive decline associated with genomic variations and neurodevelopmental disorders, whereas middle-aged and elderly patients are predominantly characterized by vascular cognitive impairment and pathological changes akin to AD. Consequently, the establishment of a stratified research model that encompasses the entire lifespan is crucial for elucidating the age-specific mechanisms of cognitive impairment related to hemodialysis (Table 1).

**Table 1:** Age-cognition-heterogeneity.

Age Group	Common mechanisms	Unique mechanisms
All ages	Inflammation and oxidative stress	–
	Dialysis hypotension	
	Uremic toxins	
≤18 years	–	Genomic variation
		Key to neurodevelopment
≥50 years	–	AD
		Vascular disease
		Dialysis hypotension

### 2.1 Children and adolescents

In pediatric populations with chronic kidney disease (CKD), there exists a notable comorbidity between neurodevelopmental disorders and cognitive deficits. It is important to highlight the presence of a distinct “window period effect” during childhood neural development, particularly during the critical phases of synaptic pruning and myelination, which occur between the ages of 2 and 12 years. During this period, metabolic disturbances associated with CKD may lead to cascading detrimental effects on the overall efficiency of brain networks. Neurodevelopmental impairments have long been recognized as a significant complication of CKD in children. Furthermore, cognitive deficits in this demographic tend to present as marginally reduced intellectual capabilities, alongside increased difficulties in short-term memory, attention regulation, and executive functioning (Harshman and Hooper, 2020). Children undergoing kidney replacement therapy, which encompasses both hemodialysis and functional kidney transplantation, tend to exhibit diminished average cognitive abilities. Furthermore, their academic performance in areas such as mathematics and literacy are also notably inferior (Kim et al., 2022). It is noteworthy that advancements in neuroimaging technology have established a foundational basis for research on cognitive decline in pediatric populations. Matsuda-Abedini et al. (2018) performed structural magnetic resonance imaging on samples obtained from individuals with CKD, including those undergoing hemodialysis, as well as control subjects. The findings indicated a reduction in white matter integrity, particularly reflected in diminished anisotropy scores within the anterior limb of the internal capsule. These results provide additional evidence that CKD in pediatric populations may exert various systemic effects on both vascular structures and cerebral function, with white matter impairment potentially serving as a contributing factor to cognitive decline. Furthermore, alterations in blood oxygenation levels within functional brain sequences, as assessed by functional magnetic resonance imaging (fMRI), can reveal irregularities in the brain networks associated with executive function (Liu et al., 2018). The existing neuroscience literature (Raichle et al., 2001; Raichle, 2015; Gusnard et al., 2001) indicates that the brain is structured into functional networks, with the default mode network (DMN) serving as a critical circuit for the regulation of attention, which may be linked to executive functioning. Given the gradual nature of neural development alongside CKD, it is imperative to concurrently investigate both neuroimaging and neurocognitive functioning in pediatric populations with CKD. Drawing from these theoretical frameworks, cognitive decline observed in children and adolescents undergoing hemodialysis demonstrates a degree of heterogeneity.

#### 2.1.1 Genomic variation

According to the theory of embryonic developmental homology, there may be a shared genetic foundation linking CKD in pediatric populations to neurocognitive disorders. Research (Verbitsky et al., 2015) indicates that around 35% of instances of congenital kidney and urinary tract anomalies are associated with pathogenic copy number variations (CNVs). These variations not only impact nephron development but may also disrupt critical neural developmental processes, including the migration of neural crest cells. It is important to highlight that there is a temporal overlap in the developmental phases of the kidneys and the cerebral cortex during the embryonic period of 4 to 8 weeks (Krieg, 2014). Drawing from these investigations, certain researchers have suggested that genomic diseases could provide a biological foundation for the association between neurocognitive disorders and CKD (Verbitsky et al., 2017). Verbitsky et al. (2017) conducted a comparative study involving children diagnosed with CKD (*n* = 31) and a control group comprising children without genomic diseases or carriers (*n* = 389). The findings revealed that the children with genomic diseases exhibited significantly poorer performance across all measures of intelligence and executive function. In a study on CNVs mutations in congenital malformations of the kidneys and urinary tract (Verbitsky et al., 2019), six loci (1q21, 4p16.1-p16.3, 16p11.2, 16p13.11, 17q12, and 22q11.2) were found to account for 65% of genomic disease pathogenic copy number variations (GD-CNVs), with the deletion of 16p11.2 being the longest leading cause of neurological abnormalities (92.5%), including neurodevelopmental delay and malformations. The absence of 22q11.2 can cause learning difficulties and intellectual disabilities, leading to an increased prevalence of a range of neurocognitive deficits (Morrison et al., 2020). A recent study (Urresti et al., 2021) has shown that the deletion and duplication of 16p11.2 not only directly affect the size of brain development, but also affect neuronal maturation, proliferation, and synaptic count. Not only that, the study also demonstrated that 16p11.2 CNV alters the ratio of neurons to progenitor cells in early neurogenesis. Through transcriptomics and proteomics, it was found that 16p11.2 CNV is dysregulated through various pathways such as neuronal migration, actin cytoskeleton, ion channel activity, synaptic related functions, and Wnt signaling transduction. The 22q11.2 region gene regulates neural crest development through TBX1, and DGCR8 deficiency leads to miRNA dysregulation and neurotransmitter metabolism affecting brain structure and function, ultimately resulting in neurodevelopmental disorders and psychiatric disorders (Purow et al., 2025).

#### 2.1.2 Developmental characteristics of the central nervous system

The establishment of functional networks within the central nervous system initiates during the embryonic phase of neural tube development and undergoes a phase of accelerated maturation postnatally, marked by the dynamic equilibrium and functional reorganization of synapses. Following birth, the quantity of synapses experiences exponential growth, potentially reaching double the adult level by the age of two. This synaptic overproduction serves as a foundational substrate for the optimization of neural circuits influenced by environmental experiences (Johnston et al., 2009). The establishment of new synaptic connections or the reinforcement of pre-existing synaptic functions can improve the communication and signal transmission among neurons, resulting in alterations in the functionality of neuronal circuits. Through mechanisms such as long-term potentiation and long-term depression, synaptic connections between neurons can be modulated, thereby facilitating processes such as memory formation, skill acquisition, and habituation (Magee and Grienberger, 2020). The strength and functionality of synapses undergo modifications during the developmental process as a result of various environmental stimuli. Additionally, these changes are meticulously regulated by a range of intrinsic factors, which encompass transcription factors, growth factors (such as brain-derived neurotrophic factor (BDNF) and insulin-like growth factor), cell adhesion molecules, and signaling molecules.

Neuroplasticity encompasses the alterations in both the structure and function of the brain across the lifespan. This phenomenon facilitates the brain’s ability to modify and adjust in response to internal or external stimuli by reorganizing its architecture, functionality, or neural connections, leading to both physiological and morphological transformations. Such a dynamic process is crucial for adapting to diverse experiences and environments from the moment of birth, and it significantly contributes to the processes of learning, memory, and recovery from brain injuries (Voss et al., 2017). Throughout the perinatal period and early childhood, the brain experiences significant and rapid growth and development, characterized by a heightened level of neural plasticity. Research (Knudsen, 2004) indicates that the brain is particularly responsive to environmental changes during this developmental period, facilitating the formation of new neural connections. It is important to highlight that neural plasticity in childhood exhibits a pronounced sensitivity to environmental factors. Additionally, systemic inflammatory markers, such as IL-6 and TNF-*α*, have been shown to inhibit neurogenesis in the hippocampal dentate gyrus, which can result in diminished synaptic efficacy related to spatial memory (Graham et al., 2021). The simultaneous presence of heightened plasticity and increased vulnerability characteristic of this developmental stage renders the central nervous system more susceptible to enduring neurodevelopmental abnormalities in the context of pathological conditions, including metabolic disorders and exposure to toxins. Some studies (Zhu et al., 2022) have shown that the abnormal nervous system function mechanism of uremic neuropathy in children involves uremic solute retention, oxidative stress, neurotransmitter imbalance and blood brain barrier destruction. In a mouse model of CKD (Bobot, 2023), indophenol sulfate activates the aromatic hydrocarbon receptor to disrupt the tight junction proteins of the blood–brain barrier (such as zonula occludens-1, occludin), causing toxins to enter the brain and inducing apoptosis of astrocytes, thereby increasing blood–brain barrier permeability and leading to memory impairment.

Numerous studies (Findlay et al., 2019; Gottlieb et al., 1987; Polinder-Bos et al., 2018; Chung et al., 2016) indicate that cerebral blood flow may diminish by 7 to 22% during hemodialysis. While certain patients may mitigate the reduction in cerebral oxygen levels and oxygenation through compensatory mechanisms that enhance oxygen delivery and utilization, it is important to recognize that extended periods of hemodialysis can lead to dysfunction in cerebral microcirculation. This dysfunction can adversely impact the metabolic microenvironment within the brain and disrupt the formation of neural networks during the critical phases of childhood development. The primary pathological alterations associated with chronic cerebral hypoperfusion encompass heightened neuroinflammation and oxidative stress, disturbances in mitochondrial function and lipid metabolism, as well as a reduction in the expression of growth factors (Washida et al., 2019). Despite the absence of direct investigations examining the correlation between diminished cerebral blood flow and brain function in infants and pediatric patients undergoing hemodialysis, researchers have utilized multimodal magnetic resonance imaging to demonstrate that within the brain’s DMN, local cerebral blood flow exhibits a more rapid increase, thereby more effectively satisfying the metabolic demands of neurons during the maturation of the DMN (Yu et al., 2023). Conversely, this suggests that a reduction in local cerebral blood flow velocity may impair the functionality of this central nervous network, consequently impacting cognitive abilities and associate behavioral performance. Thus, in infants and children, the disruption of microcirculation and metabolic coupling resulting from hemodialysis may contribute to long-term cognitive deficits by interfering with neural networks. Furthermore, a recent case–control study (van der Plas et al., 2025) has revealed significant age-related disparities in neurodevelopment among pediatric patients with CKD when compared to their healthy counterparts, with a notable reduction in cerebellar volume linked to cognitive impairments and diminished renal function. Although the precise mechanisms through which hemodialysis directly influences neurological development in children remain unclear, the distinctive nature of brain development in this population, along with the occurrence of neurocognitive disorders following hemodialysis, provides a theoretical framework for future investigations.

### 2.2 Elderly patients (≥ 50 years old)

The elevated prevalence of AD and vascular cognitive impairment among elderly individuals receiving hemodialysis can be attributed to the interplay of various pathophysiological mechanisms. Epidemiological studies indicate that conditions such as hypertension, diabetes, atrial fibrillation, carotid artery disease, and dyslipidemia are frequently linked to ESRD (Toyoda and Ninomiya, 2014). Collectively, these factors may serve as dual contributors to cognitive impairment, encompassing age-related vascular lesions and the pathophysiological stress associated with hemodialysis.

#### 2.2.1 Independent contribution of vascular diseases

A recent systematic review (Cao et al., 2023) investigating the risk factors associated with cognitive impairment in patients undergoing maintenance hemodialysis identified several primary risk factors, including advanced age, female gender, hypertension, diabetes, a history of cerebrovascular accidents, and variability in systolic blood pressure. Notably, cardiovascular diseases, such as atherosclerosis and heart failure, exhibited a particularly strong correlation with cognitive impairment. Furthermore, it is important to highlight that the risk factors associated with cardiovascular disease—namely hypertension, diabetes, and hyperlipidemia—demonstrate a dose-dependent adverse effect on cognitive function, with the associated risk being significantly elevated compared to that of the general population (Cheung et al., 2000; Cheung et al., 2004; Karakizlis et al., 2022). This phenomenon is particularly pronounced in individuals with ESRD. In comparison to the general elderly population, patients with ESRD exhibit a higher incidence of cerebrovascular events, including ischemic strokes and transient ischemic attacks. Furthermore, they are also more susceptible to subclinical cerebrovascular abnormalities, such as small vessel infarctions, white matter hyperintensities (WMHs), and microbleeds (Drew et al., 2013; Drew et al., 2017a). An examination of the pathological and physiological mechanisms reveals that arteriosclerosis, reduced microvascular density, and compromised autonomic regulation of cerebral blood flow are fundamental pathways contributing to vascular cognitive impairment in the elderly population (Amier et al., 2021). Atherosclerosis results in structural impairment of small cerebral blood vessels, characterized by endothelial dysfunction and smooth muscle hyperplasia, which contributes to hypoperfusion of white matter and accelerates cognitive deterioration. Furthermore, the process of hemodialysis itself exacerbates vascular injury through various mechanisms, including hemodynamic stress, such as hypotension experienced during the procedure, and endothelial dysfunction. Numerous imaging studies have demonstrated that cerebral blood flow may diminish during hemodialysis, with this fluctuation exhibiting a significant negative correlation with declines in cognitive function scores (Findlay et al., 2019; Polinder-Bos et al., 2018). The swift decrease in systolic blood pressure during dialysis in elderly patients, attributed to compromised autonomic cardiovascular regulation alongside atherosclerosis and arteriosclerotic vascular conditions, renders these individuals more susceptible to the effects of abrupt reductions in cerebral blood flow. This interplay between diminished cerebral perfusion and altered brain osmotic pressure contributes to ischemic changes in white matter, thereby intensifying cognitive deficits (Khatri and Davenport, 2024). Intermittent fluctuations in blood pressure during dialysis, particularly episodes of hypotension, can cause significant alterations in cerebral perfusion pressure. This phenomenon is associated with an annual increase of 5.3% in the volume of white matter hyperintensities (Findlay et al., 2019). Furthermore, the recurrent cyclic stress associated with hemodialysis may contribute to ischemic encephalopathy, as the cerebral blood flow experiences repeated reductions during the procedure, potentially exacerbating cognitive decline (Polinder-Bos et al., 2018).

#### 2.2.2 Accelerated progression of AD

Given that advanced age is a prevalent risk factor for both AD and ESRD, a comorbid relationship between these two conditions is frequently observed. Epidemiological studies (Chen et al., 2023) indicate that the incidence of mild cognitive impairment and dementia among patients undergoing hemodialysis is markedly elevated compared to that of the general population. Empirical studies have indicated that the dysregulated activation of the renin-angiotensin system within the central nervous system, coupled with neuroinflammatory responses—characterized by the proliferation of microglia and astrocytes—and the impairment of the blood–brain barrier, constitute the fundamental mechanisms driving the pathological advancement of AD (Ma et al., 2023; Zheng and Wang, 2025). In contrast to the peripheral renin-angiotensin system, the central renin-angiotensin system operates via a distinct pathway. In this system, renin catalyzes the conversion of angiotensinogen into angiotensin I (Ang-I), which is subsequently transformed into angiotensin II (Ang-II) by angiotensin-converting enzyme (ACE) within neurons and astrocytes. Ang-II then interacts with angiotensin II receptor type 1 (AT1R) and angiotensin II receptor type 2 (AT2R) receptors located on the surfaces of microglia, astrocytes, and neurons, initiating a series of events that lead to oxidative stress, neuroinflammation, and neuronal apoptosis (Rivas-Santisteban et al., 2021). Preclinical investigations (Nakagawa et al., 2017) have further substantiated that CKD can exacerbate cognitive decline in murine models of AD. Specifically, the presence of Ang-I receptors appears to heighten the vulnerability of Alzheimer’s-afflicted mice to disruptions of the blood–brain barrier and cognitive impairments induced by oxidative stress associated with CKD. In patients exhibiting mild cognitive impairment or diagnosed with AD the proliferation of astrocytes and microglia, along with the resultant inflammatory response, may accelerate neuronal dysfunction and cognitive decline in the context of CKD. Consequently, for hemodialysis patients with AD, the increased presence of astrocytes and inflammatory responses in the central nervous system, coupled with the overactivation of the renin-angiotensin system, may synergistically contribute to the worsening of cognitive impairment. This phenomenon may also represent a potential mechanism through which ACE inhibitors and Ang-II receptor antagonists could mitigate neuronal dysfunction and inflammation, thereby reversing cognitive deficits.

#### 2.2.3 The key pathway of synergistic effect: blood–brain barrier

At the capillary level, the endothelium serves as a crucial element of the blood–brain barrier, and dysfunction of the endothelium may be linked to cognitive decline (Wu et al., 2023). Within the spectrum of endothelial-related biomarkers, four distinct types are identified, each serving unique functions: intercellular adhesion molecule-1 (ICAM-1), vascular cell adhesion protein-1 (VCAM-1), angiopoietin-2 (AGPT2), and syndecan-1. These biomarkers are associated with the activation of endothelial cells (Freire de Medeiros et al., 2020). Preliminary studies have established that the disruption and heightened permeability of the blood–brain barrier significantly contribute to cognitive dysfunction, not only in the context of neurodegenerative disorders but also in the mechanisms underlying cognitive impairment associated with renal diseases (Kadry et al., 2020; Nation et al., 2019). Currently, numerous studies (Angermann et al., 2022; Shoji et al., 2022; Wang et al., 2023; Kelly et al., 2024) have established a correlation between alterations in vascular function and cognitive performance in individuals with CKD undergoing hemodialysis. A prospective cohort study (Libório et al., 2024) investigating endothelial-related biomarkers and cognitive decline in this patient population revealed that syndecan-1 serves as a predictor of cognitive decline indicators among hemodialysis patients. This finding suggests that certain vascular endothelial factors may not only reflect cognitive impairment in this demographic but also function as predictive markers, thereby providing a theoretical basis for the identification of novel therapeutic targets in future research.

#### 2.2.4 The sequence of physiological changes associated with hypotension caused by dialysis and its link to neurodegeneration

In the elderly population, dialysis-induced hypotension (IDH), which occurs in 30 to 40% of cases, accelerates neurodegeneration through a series of events: “decreased cerebral blood flow → endothelial damage → disruption of the blood–brain barrier → neuroinflammation → oxidative stress → neurodegeneration.” The decline in vascular reserve is particularly pronounced in older patients. Specifically, when systolic blood pressure drops by 20 mmHg or more, or falls below 90 mmHg during dialysis, cerebral blood flow can decrease by 7 to 22%. Due to factors like atherosclerosis and impaired autonomic regulation, elderly patients struggle to maintain adequate cerebral perfusion, particularly in cognitive areas such as white matter and the hippocampus. A sudden drop in cerebral blood flow can harm the endothelial cells of cerebral blood vessels through mechanical and metabolic stress, resulting in reduced vasodilator factors and increased contractile factors. This also disrupts the connections between endothelial cells, raising the permeability of the blood–brain barrier, which can be indicated by elevated levels of endothelial injury markers like syndecan-1. Once the blood–brain barrier is compromised, uremic toxins and inflammatory factors can enter the brain, hindering neuronal synaptic plasticity and activating microglia and astrocytes, which leads to neuroinflammation, including excessive phosphorylation of tau protein. Ultimately, reduced cerebral perfusion and neuroinflammation trigger oxidative stress, resulting in mitochondrial dysfunction, decreased synaptic density, and compromised white matter integrity, which can cause neuronal apoptosis in key cognitive areas and accelerate cognitive decline. These concepts have been supported by subsequent clinical research. The KIDBRAIN (Cohort Study of Morphological Changes of the Brain by MRI in Chronic Kidney Disease Patients) study by Cedeño et al. (2021) found that dialysis hypotension is linked not only to structural brain damage but also to cognitive decline. Notably, even asymptomatic dialysis hypotension can indirectly impair cognition by damaging white matter and the hippocampus, while symptomatic hypotension has a direct impact on cognitive function. Additionally, repeated mild episodes of dialysis-induced hypotension (such as a systolic blood pressure drop of 20 mmHg or more) can cause more significant long-term damage to brain structure than a single severe episode.

## 3 Precision intervention strategy based on lifecycle

### 3.1 Children and adolescents: neurodevelopmental protection and prenatal screening intervention

In light of the defining features of critical periods in neurological development among pediatric patients, it is essential for clinical interventions to achieve a balance between safeguarding the integrity of the blood–brain barrier and preserving synaptic plasticity.

#### 3.1.1 Neurodevelopmental support therapy

In recent years, neural stem cells (NSCs) have emerged as a focal point of investigation within the domain of regenerative medicine, attributed to their distinctive capabilities for self-renewal and multi-directional differentiation (Beattie and Hippenmeyer, 2017; Wang et al., 2025). Fundamental research has demonstrated that NSCs can effectively enhance neurogenesis and tissue remodeling in the central nervous system by selectively differentiating into and replenishing damaged neurons, releasing neurotrophic factors (like BDNF and glial cell-derived neurotrophic factor), and activating natural repair mechanisms (Wang et al., 2025). However, it is important to note that the use of NSC transplantation in pediatric patients undergoing hemodialysis is still largely theoretical, with no preclinical or clinical studies conducted specifically for this group. The neuroprotective effects, safety, and ideal timing for intervention remain uncertain. Current research primarily focuses on neurodevelopmental disorders in non-dialysis children, such as cerebral palsy, and often involves small sample sizes. For instance, a study (Bansal et al., 2016) involving children with congenital brain injuries indicated that NSC transplantation could enhance motor function in some cases, but did not assess cognitive outcomes, and there are significant differences in the underlying pathology between this group and children on hemodialysis, such as exposure to uremic toxins and abnormal cerebral blood flow. Consequently, the potential cognitive benefits of NSC transplantation for pediatric dialysis patients can only be hypothesized based on its known neural repair properties, and further research is needed to confirm its relationship with cognitive improvement.

To advance research on NSC transplantation in pediatric hemodialysis patients, several feasibility issues must be addressed: (1) Development of animal models: It is essential to create a model that mimics dialysis-related cognitive impairment in children (for example, a model of dialysis-induced white matter injury in young rats) to assess the effects of NSC transplantation on synaptic plasticity and default mode network function; (2) Timing of transplantation: It is crucial to determine whether NSC transplantation during critical periods of neural development (such as ages 2–12) can reverse existing cognitive impairments or merely prevent new injuries; (3) Safety evaluation: It is necessary to investigate how the immune status of dialysis patients (including immune suppression from chronic inflammation) affects the survival of NSCs and whether there is an increased risk of complications like infections and vascular embolism following transplantation.

#### 3.1.2 Gene–environment synergistic intervention

Over the past 10 years, the role of genetic factors in kidney health has become increasingly apparent, with more than 600 genes identified as being associated with monogenic kidney disorders (Rasouly et al., 2019). Fetal abnormalities are observed in approximately 3% of pregnant women and are responsible for around 20% of all perinatal mortality. These abnormalities encompass a range of chromosomal and genetic disorders, including aneuploidy, CNVs, and pathogenic variations specific to certain genes (Sotiriou et al., 2024; Vora and Hui, 2018). While a preliminary link between CNVs and synaptic plasticity has been identified, the impact of particular types of CNVs (like pathogenic microdeletions and microduplications) on synaptic-related molecular pathways (including Wnt signaling transduction) requires additional confirmation. Utilizing single-cell sequencing alongside organoid models could help identify the specific targets of CNVs within the blood–brain barrier synaptic axis. For children with pathogenic CNVs, it is essential to implement a three-tiered prevention and management framework that encompasses prenatal diagnosis, dialysis monitoring, and stem cell intervention. In recent years, established protocols for prenatal diagnosis have involved the utilization of fetal ultrasound to detect pathogenic anomalies, subsequently followed by standard karyotype analysis and/or chromosome microarray analysis utilizing DNA samples obtained from amniotic fluid, chorionic villi, or umbilical cord blood. The advent of whole exome sequencing (WES) and whole genome sequencing (WGS) has markedly enhanced diagnostic accuracy (Jelin and Vora, 2018; Kim et al., 2024). In cases where pathogenic genes are identified in the kidneys and brain through prenatal diagnosis, early intervention may be implemented to mitigate the risk of postnatal diseases, thereby lessening the burden on both families and society.

### 3.2 Elderly patients (≥ 50 years old)

The fundamental process underlying cognitive impairment in elderly patients undergoing hemodialysis is attributed to the interplay between vascular disease and neurodegeneration. This necessitates the implementation of a multi-targeted intervention strategy.

#### 3.2.1 Control of vascular diseases

For patients undergoing maintenance hemodialysis who present with hypertension, diabetes, arteriosclerosis, and other underlying health conditions, enhancing the management of the primary disease is essential for the prevention of complications. The etiology of hypertension in this population is multifaceted. In addition to intrinsic factors such as age, genetic predisposition, and detrimental lifestyle choices, hypertension is also associated with volume overload, heightened arterial stiffness, hyperactivity of the sympathetic nervous system, excessive activation of the renin-angiotensin-aldosterone system (RAAS), endothelial dysfunction, and the administration of erythropoietin (EPO) (Bansal et al., 2023). In the context of cognitive impairment associated with hypertension, it is advisable to prioritize antihypertensive medications that possess neuroprotective properties. Specifically, angiotensin receptor blockers and ACE inhibitors are recommended, as they can mitigate the excessive activation of the renin-angiotensin system. These agents not only effectively manage blood pressure but also contribute to the reduction of cerebrovascular disease progression and the alleviation of cognitive deficits (Kobayashi et al., 2014). Furthermore, given that cerebrovascular diseases, including cerebral small vessel disease and asymptomatic infarction, represent significant risk factors for cognitive impairment in patients undergoing maintenance hemodialysis, it is advisable to adopt a stratified screening approach that is informed by standard antihypertensive treatment protocols. Initially, for patients with CKD (CKD) who have not yet commenced dialysis, a baseline cognitive evaluation should be conducted utilizing the Montreal Cognitive Assessment Scale (MoCA) or the Mini-Mental State Examination (MMSE) (Tiffin-Richards et al., 2014). Furthermore, biannually, a collaborative neurology specialist will perform neuroimaging assessments, including MRI-DWI evaluations to detect cerebral microbleeds and FLAIR sequence imaging to identify white matter lesions, in addition to testing for cerebral blood flow autoregulation function. Lastly, for individuals diagnosed with mild cognitive impairment through screening processes, it is essential to assess amyloid deposition, utilizing techniques such as Amyloid-*β* Position Emission Tomography(Aβ-PET)imaging, and to implement a multidisciplinary intervention strategy (Drew et al., 2019).

#### 3.2.2 Targeted regulation of AD

In patients undergoing hemodialysis, the presence of uremic toxins, alterations in cerebral blood flow, and various dialysis-related factors may contribute to an increased permeability of the blood–brain barrier, subsequently resulting in cognitive decline (Hernandez et al., 2022; Bobot et al., 2020; Rosner et al., 2022). Recent research and case reports (Kitaguchi et al., 2018; Kitaguchi et al., 2019; Kitaguchi et al., 2021; Saito et al., 2020) have indicated that hemodialysis treatment can transiently decrease the amyloid burden in the brain by facilitating the clearance of Amyloid-beta (Aβ) and tau proteins from peripheral circulation. This observation has prompted the hypothesis of employing hemodialysis as an *in vitro* intervention strategy for AD. Current observational studies suggest that patients with AD undergoing hemodialysis may experience short-term enhancements in cognitive symptoms as a result of regular dialysis sessions.

However, there is significant controversy surrounding the efficacy of this intervention, with the core question being: Does hemodialysis-mediated Aβ clearance truly alter the natural course of AD, or does it merely cause transient changes in Aβ levels without affecting neurodegenerative progression? On one hand, preliminary evidence suggests that hemodialysis can transiently reduce peripheral Aβ and tau levels, and some imaging data even indicate a subsequent decrease in brain Aβ burden (Kitaguchi et al., 2018; Kitaguchi et al., 2019; Kitaguchi et al., 2021; Saito et al., 2020). This has led to the hypothesis that hemodialysis may promote the efflux of Aβ from the brain to the periphery through a concentration gradient. However, these observations are limited by short follow-up periods (typically < 6 months) and a lack of long-term data on tau phosphorylation or neurodegenerative changes (such as hippocampal atrophy)—which are hallmark features of AD progression.

On the other hand, the repeated hemodynamic stress caused by hemodialysis, particularly intradialytic hypotension, may counteract the potential benefits of Aβ clearance. As described in section 2.2.4, dialysis-induced hypotension triggers a cascade of events: “cerebral hypoperfusion → endothelial damage → blood–brain barrier disruption → neuroinflammation → excessive tau phosphorylation,” which is consistent with the core pathological processes of AD. This dual effect creates a paradox: while hemodialysis may acutely reduce Aβ levels, it may simultaneously accelerate the neurodegenerative cascade through ischemic injury and neuroinflammation, ultimately failing to alter the natural trajectory of AD.

Further complicating the causal relationship is the difficulty of distinguishing AD-specific pathological progression from hemodialysis-related neurological damage in existing studies. For example, the annual increase in white matter hyperintensity volume (up to 5.3%) observed in elderly hemodialysis patients (Findlay et al., 2019) overlaps with the imaging features of vascular cognitive impairment and AD-related small vessel disease, making it difficult to attribute cognitive decline solely to AD progression or dialysis-induced vascular injury. Additionally, the natural course of AD is characterized by the slow and progressive accumulation of Aβ plaques and neurofibrillary tangles, whereas hemodialysis-induced changes in Aβ levels are acute and may not be sufficient to impact this chronic process. Therefore, the transient reduction in Aβ following hemodialysis is unlikely to translate into effective slowing of cognitive decline unless it persists for a period comparable to the timeline of AD pathology.

Therefore, it is still uncertain if the overall advantage of hemodialysis on cognitive impairment related to AD surpasses the cognitive deterioration seen in the disease’s natural progression. Addressing this important scientific issue urgently calls for carefully designed prospective studies that monitor Aβ/tau changes, neurodegenerative indicators, and cognitive performance over an extended period to better understand the spatial and temporal connection between hemodialysis and the pathological development of AD.

## 4 Mode selection of hemodialysis

Research indicates that patients with cognitive impairment experience considerable deficits in executive function, attention, and episodic memory. Furthermore, the rate of cognitive decline in these individuals is estimated to be 3 to 5 times greater than that observed in the general elderly population (Kanbay et al., 2025; Zhang et al., 2024). This accelerated cognitive deterioration has direct implications for adherence to dialysis, the safety of medication management, and the overall burden on family and social networks. More critically, these cognitive challenges are associated with a markedly poorer long-term prognosis, resulting in an increased risk of disability and mortality among this patient population (Gela et al., 2021; McAdams-DeMarco et al., 2018). Murray et al. (2006) conducted an evaluation of 374 patients undergoing hemodialysis and discovered that merely 13% exhibited normal cognitive function. In a related study (Sarnak et al., 2013), it was observed that hemodialysis patients performed worse on various neurocognitive assessments compared to the general population, and they also demonstrated a markedly elevated prevalence of cognitive impairment. Previous research indicates that with advancing age, there is a corresponding increase in clinical weakness scores and comorbidities, alongside a decrease in active energy expenditure. This is often accompanied by a diminished thirst drive and weight gain during dialysis. Notably, the production rate of uremic toxins tends to be lower in elderly patients. Consequently, it is imperative that dialysis prescriptions for older patients differ from those prescribed for younger individuals (Hendra et al., 2022). For hemodialysis patients, particularly across varying age demographics, the implementation of an optimal hemodialysis modality tailored to individual needs may significantly mitigate the progression of cognitive impairment.

### 4.1 Incremental dialysis

Incremental dialysis is characterized as a blood dialysis approach that leverages residual renal function to diminish the intensity of dialysis treatment. This method typically results in a frequency of dialysis sessions that is less than three times per week, with each session lasting 4 h (Khatri and Davenport, 2024). While residual renal function serves as the primary factor influencing incremental dialysis, it is essential to also take into account patient adherence to fluid and sodium restrictions, dietary guidelines, levels of physical activity, and energy expenditure (Rhee et al., 2017; Sridharan et al., 2017). Inadequate management of these factors may result in extended dialysis duration, an increase in the frequency of dialysis sessions, and heightened metabolic waste production due to increased physical activity, thereby necessitating more frequent dialysis treatments. Relevant observational studies (Elgendy et al., 2022) indicate that incremental dialysis may be more effective in preserving residual renal function, which could subsequently enhance the quality of life and cognitive assessment scores in elderly patients undergoing hemodialysis. Consequently, for this demographic, the selection of incremental dialysis tailored to individual circumstances may lead to improvements in cognitive function. However, further prospective studies are necessary to validate these findings and investigate the underlying mechanisms.

### 4.2 Low temperature dialysis

A substantial body of prior research (Selby and McIntyre, 2006; Eldehni et al., 2015; Selby et al., 2006) indicates that low-temperature dialysis offers significant advantages not only for renal function but also for other organs, including the brain, where it may mitigate the progression of white matter damage. While these initial studies did not explicitly examine the effects of low-temperature dialysis on cognitive decline, they established a foundational basis for subsequent investigations in this area. In comparison to conventional dialysis, the utilization of colder dialysate (ranging from 34 to 35 degrees Celsius) has been demonstrated to mitigate hypotension during the dialysis process by inhibiting systemic vasodilation (Mustafa et al., 2016). Research indicates that dialysis-induced hypotension may impact approximately 30 to 40% of patients undergoing hemodialysis, leading to recurrent ischemic injury in various organs, including the brain, which subsequently influences cognitive function (Buchanan et al., 2017; Odudu and McIntyre, 2016). In light of the COVID-19 pandemic, a feasibility randomized controlled trial investigating the cognitive effects of low-temperature dialysis on patients with ESRD yielded results that did not align with the initial hypothesis. Specifically, it was anticipated that patients undergoing routine hemodialysis with cooled dialysis fluid at 35 °C would experience less cognitive decline and an improved quality of life compared to those receiving standard temperature dialysis fluid at 36.5 °C. Nevertheless, the study revealed that participants in the low-temperature dialysis group exhibited enhanced dialysis tolerance, a finding that may inform the design of more definitive trials in this area in the future (Dasgupta et al., 2024).

### 4.3 High-dose hemodiafiltration

High-dose hemodiafiltration represents a significant advancement in renal replacement therapy, bolstered by a substantial body of evidence-based medical research that underscores its clinical benefits. Prospective randomized controlled trials and meta-analyses (Maduell et al., 2017; Vernooij et al., 2024; Strippoli and Green, 2024) consistently demonstrate that high-dose hemodiafiltration is more effective than conventional high-throughput hemodialysis. This enhanced efficacy is attributed to a dual mechanism: firstly, the utilization of a high-throughput capillary dialyzer that preserves an optimal surface area to blood flow ratio; and secondly, the optimization of ultrafiltration flow rates to attain a filtration fraction of 30 to 35%, alongside the development of anticoagulant regimens tailored to individual patient characteristics (Canaud et al., 2024). The recent evaluation of the clinical review system (Canaud et al., 2025) indicates that, in comparison to conventional high-throughput hemodialysis, high-dose hemodiafiltration not only enhances the rate of solute clearance but also markedly improves patient quality of life through the modulation of various inflammatory markers. Furthermore, it may result in favorable alterations in neurological function metrics, including enhancements in cognitive function. In 2024, the CONVERCE study (Rose et al., 2024) provided additional evidence that high-dose hemodiafiltration significantly enhances patients’ quality of life and decreases all-cause mortality. Notably, its influence on cognitive function improvement is particularly remarkable. Furthermore, it is important to highlight that the clinical advantages of high-dose hemodiafiltration demonstrate universal characteristics across different age groups. Research conducted in pediatric populations indicates that this technology has a beneficial impact on the growth and development of children (Shroff et al., 2019). While current research frequently assesses survival rates and cardiovascular outcomes using intermediate indicators, including oxidative stress and inflammatory markers, there is emerging evidence indicating that high-capacity hemodialysis filtration may possess mechanisms that enhance cardiovascular health and neurocognitive functions (Shroff et al., 2019). It is important to highlight that the current evidence linking high-dose hemodiafiltration to cognitive benefits primarily stems from observational studies and randomized trials that do not focus on cognitive outcomes. As a result, we can only confirm a correlation between the two, without establishing a causal link. Several potential confounding factors in these studies may influence the interpretation of the findings. First, there are variations in baseline cognitive function: patients undergoing high-capacity hemodialysis filtration often have a higher cognitive reserve (e.g., greater education and cognitive scores) or may opt for this treatment due to more stable health conditions (e.g., fewer comorbidities), which could lead to a slower cognitive decline that is not solely attributable to high-dose hemodiafiltration. Second, the effect on residual renal function may play a role: better residual renal function could help delay cognitive decline by reducing the buildup of uremic toxins and maintaining more stable hemodynamics (e.g., lower rates of hypotension during dialysis). However, existing research has not fully eliminated this mediating effect. Additionally, the follow-up period in current studies is often limited to 1–3 years, making it challenging to account for the natural fluctuations in cognitive function. For instance, short-term improvements might be linked to toxin clearance, but it remains uncertain whether these improvements are sustained in the long term.

While there is presently a lack of specialized research addressing cognitive function in individuals with ESRD, the progression of research paradigms exhibits notable characteristics at various stages. The growing body of evidence regarding key endpoint indicators, such as enhanced survival rates and the mitigation of cardiovascular and cerebrovascular complications through advanced blood dialysis filtration techniques, has positioned neurocognition as an emerging area of investigation within this technological domain. This transition in research emphasis aligns with the principles of evidence-based medicine and suggests a paradigm shift in blood purification technology from merely serving as a replacement therapy to encompassing a more holistic approach to patient management.

## 5 Conclusion

**Figure 1:**
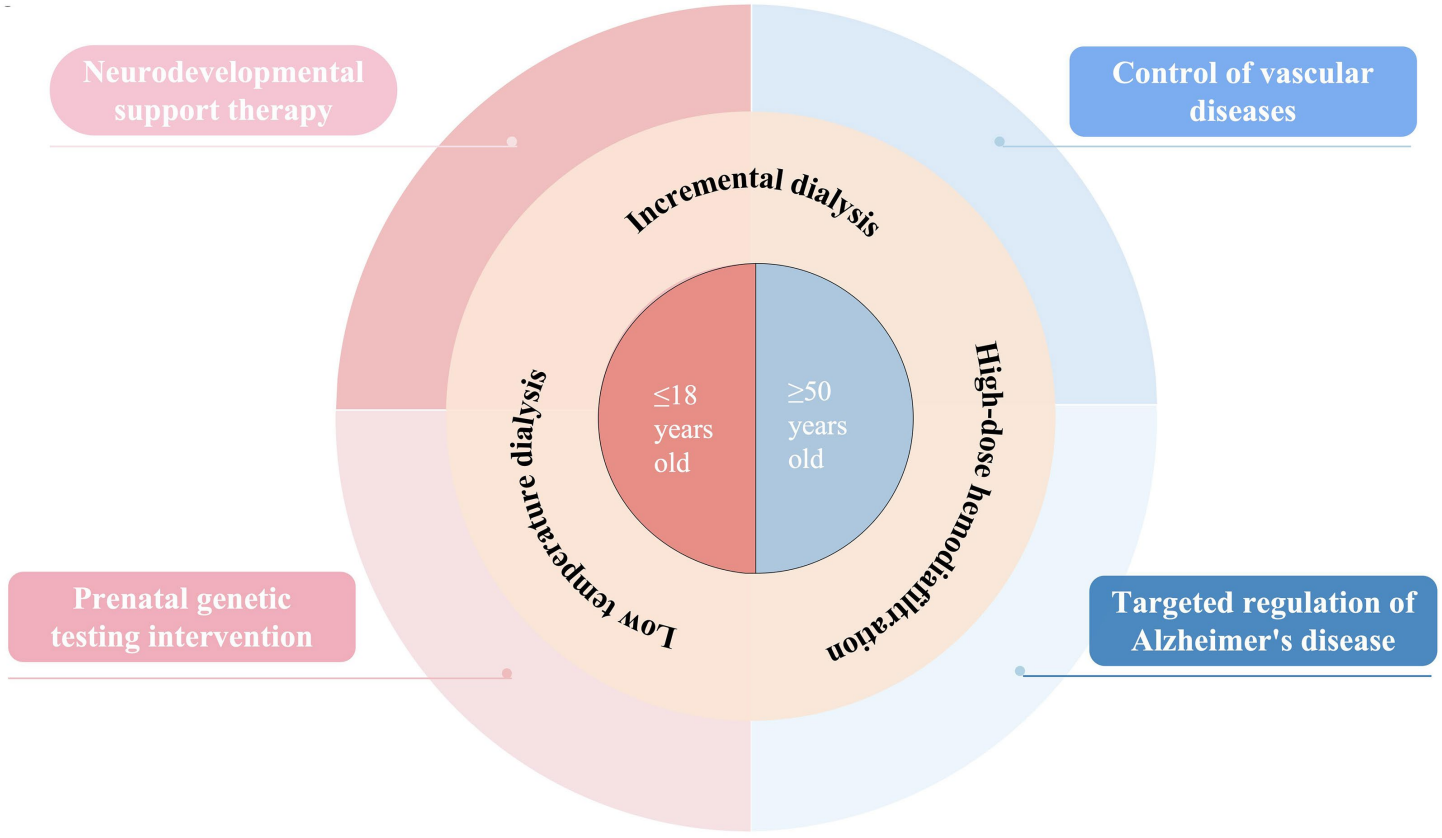
Precise intervention.

This research systematically uncovered the age-specific mechanisms behind cognitive decline in patients undergoing hemodialysis and developed an innovative intervention system tailored to different life stages (Figure 1). By conducting a thorough analysis of two distinct groups—children and the elderly—we discovered that cognitive impairment in pediatric patients primarily arises from their heightened vulnerability during critical neural development phases. During this time, the blood–brain barrier becomes more permeable, allowing uremic toxins to penetrate the central nervous system, which damages white matter integrity; neuroimaging reveals a notable reduction in anisotropy scores. Additionally, genetic factors, such as pathogenic copy number variations, worsen cognitive deficits by disrupting kidney-brain co-development, while a 7 to 22% reduction in cerebral blood flow during dialysis significantly affects the formation of neural networks. In contrast, cognitive decline in older patients exhibits more intricate pathological characteristics. Our findings indicate that cerebral small vessel disease and recurrent hypotension during dialysis contribute to chronic cerebral hypoperfusion, with imaging data showing an annual increase of up to 5.3% in the volume of white matter hyperintensities. The uremic environment accelerates changes similar to those seen in AD, promoting the deposition of Aβ and the phosphorylation of tau protein, while excessive activation of the renin-angiotensin system intensifies neuroinflammatory responses. Importantly, the integrity of the blood–brain barrier is crucial in this context, and elevated levels of specific biomarkers like syndecan-1 can not only indicate the extent of endothelial damage but also forecast the trajectory of cognitive decline.

Based on these results, we have created intervention strategies designed for various age groups. For children, the emphasis is on neuroprotection and early intervention, with NSC transplantation technology showing encouraging potential. This approach facilitates synaptic remodeling through mechanisms like the release of BDNF and animal studies have demonstrated its ability to repair white matter damage. Additionally, combining prenatal whole genome sequencing with perinatal intervention is anticipated to lower the risk of cognitive impairment from the outset. However, future research should concentrate on translating evidence for targeted interventions, such as moving from basic research to preclinical trials for pediatric neural stem cell transplantation, which requires validation of its cognitive protective benefits through specific models for dialysis patients. For elderly patients, a multi-target combined intervention is necessary, which includes using renin-angiotensin system inhibitors alongside low-temperature dialysis to stabilize cerebral blood flow. There is also a need to investigate the potential benefits of high-dose hemodiafiltration in removing peripheral Aβ. Future interventions should aim to interrupt the pathway of dialysis hypotension leading to cerebral blood flow irregularities and neurodegeneration. Furthermore, by addressing the decline in vascular reserve function in older patients, optimizing blood pressure management strategies during dialysis can help slow cognitive decline.

High-dose hemodiafiltration has demonstrated notable protective benefits across different age groups, effectively removing large molecule uremic toxins and significantly lowering inflammatory markers. The incremental dialysis approach offers a new way to enhance the quality of life for elderly patients by maintaining their residual kidney function. Current clinical evidence indicates that the successful integration of technological advancements with underlying pathological mechanisms is crucial for improving patient outcomes. However, it is important to note that the link between high-dose hemodiafiltration and improvements in cognitive function still needs more thorough investigation. The advantages indicated by existing observational studies may be affected by confounding variables, such as initial cognitive differences and residual kidney function, and their direct impact on slowing cognitive decline must be validated through randomized controlled trials focused on cognitive function as the main outcome. Future studies should create specialized cohorts to better understand the actual effects of high-dose hemodiafiltration on cognitive protection, taking into account baseline cognitive levels, adjustments for residual kidney function, and other confounding factors like comorbidities.

In the future, creating a multi-center research group that spans the entire lifecycle and incorporates diverse data from genomics, proteomics, and neuroimaging will enable the dynamic tracking of cognitive function changes. On a technical front, the emphasis will be on critical challenges like enhancing dialysis clearance efficiency and increasing tolerance to low-temperature dialysis. The adoption of precision medicine principles will pave the way for tailored treatment options. Furthermore, investigating non-dialysis dependent support methods, such as managing gut microbiota to decrease toxin levels, could offer innovative approaches for cognitive protection.

The groundbreaking importance of this research is in the creation of an age-based management system that focuses on pathological mechanisms, moving away from the conventional “one size fits all” treatment approach to more targeted interventions. By implementing early interventions in childhood to lower the risk of long-term disabilities, employing multi-target therapies in older adults to slow the progression of dementia, and enhancing dialysis technology to improve quality of life, we are shifting kidney disease treatment from merely replacing organ function to maintaining neurological function. Future studies should aim to develop longitudinal cohorts across different age groups and examine the relationship between hemodialysis and neurodegenerative diseases over time. Through interdisciplinary collaboration and the application of precision medicine technologies, we anticipate a significant advancement from “pathological relief” to “neural function remodeling,” paving the way for a new era in cognitive health management for patients undergoing hemodialysis.
